# Supplementary material for: Visual processing oscillates differently through time for adults with ADHD
Source: PLoS One. 2025 Sep 2;20(9):e0310605. doi: 10.1371/journal.pone.0310605 (PMC12404544; doi:10.1371/journal.pone.0310605)
Supplement: Attachment — Submitted filename: Review Comments to the Author.pdf [file pone.0310605.s003.pdf]

**Dear Authors,**

Following the review of successive versions of this manuscript, it is noted that the work has adequately addressed the issues raised by the reviewers. This study represents a relevant contribution to the understanding of ADHD and to the field of cognitive and computational neuroscience. The work is situated at the intersection of perceptual psychophysics, computational neuroscience – particularly predictive processing models and oscillatory dynamics – and the emerging area of digital biomarkers for neurodevelopmental disorders. The adopted approach combines the random temporal sampling (RTS) methodology with machine learning (SVM) classification to investigate the temporal dynamics of visual perceptual processing, revealing aspects of brain function in adult ADHD that may not be captured by more static approaches.

## Strengths and In-depth Theoretical Integration

1. **RTS as a Tool to Investigate Predictive Dynamics:** The RTS methodology, generating time-frequency CIs, functions as a psychophysical tool to investigate the *temporal precision* and *efficiency of information utilization* under uncertainty (noise). This aligns with contemporary theoretical frameworks that view the brain as a predictive inference system (e.g., Friston, 2005; Hohwy, 2013), constantly confronting internal predictions (priors) with afferent sensory evidence to compute prediction errors. Alterations in this process have been proposed in neurodevelopmental disorders (e.g., Gonzalez-Gadea et al., 2015; Samengo et al., 2022). The fluctuations in visual processing efficiency captured by the CIs in this study (Figures 2, 3) can be interpreted as a behavioral manifestation of the stability (or instability) with which the ADHD neural system generates, weights, and updates these predictions. The frequency-domain analysis (pp. 7-8) aligns with the view of neural oscillations as a substrate for *timing*, sensory *gating*, and inter-area communication (Buzsáki, 2006; Klimesch, 2012). The methodological rigor employed (pp. 7-10) confers robustness to the current findings.

2. **The Temporal Phenotype of ADHD – Oscillations, Filtering, and Arousal:** The high accuracy of the SVM classifier in distinguishing ADHD vs. NT (91.8%; p. 12) suggests a consistent and potentially replicable *processing signature* in ADHD (high intra-group coherence; p. 18), despite its clinical heterogeneity (Faraone et al., 2015; Luo et al., 2019). This signature may reflect systematic alterations in the parameters governing the weighting between predictions and sensory error, or in the efficacy of mechanisms for filtering irrelevant stimuli – a concept related to latent inhibition, a filtering process that may be altered in ADHD (e.g., Micoulaud-Franchi et al., 2015). The importance of low frequencies (5-15 Hz, alpha/theta; p. 13) as key discriminators is relevant, as alpha/theta rhythms are linked to inhibitory *gating* and anticipatory *timing* (Haigh & Buckby, 2024; Kannen et al., 2024; Klimesch, 2012). The observed pattern may indicate a deficit in these mechanisms, resulting in greater vulnerability to distraction. It can be hypothesized that such processing instability and low threshold for distraction connect to the need for movement, viewed as a compensatory strategy to regulate cortical *arousal* in ADHD (Hartanto et al., 2016; Sikström & Söderlund, 2007), possibly via modulation of ascending systems such as the ARAS.
3. **Medication Effects as Neuromodulatory Recalibration:** The ability to classify medication status (91.3%; p. 14) with a distinct set of spectral characteristics (Figure 6) is a relevant finding. It suggests that psychostimulants may *recalibrate* the predictive system, perhaps by strengthening *priors* or optimizing sensory gain control via DA/NA systems that modulate thalamo-cortical circuits and ascending activating systems (e.g., Mamiya et al., 2021; Sara & Bouret, 2012; Volkow et al., 2011). The apparent "normalization" of the CI pattern in medicated individuals (Figure 5 vs. Figure 2a) may represent the behavioral correlate of this optimization (p. 19), consistent with some findings on long-term effects of stimulants (Konrad et al., 2007; Swanson et al., 2011). The difference in discriminatory frequency profiles (p. 14) may indicate distinct targets: central instability versus adaptive gain/precision adjustments.

4. **Convergence with Digital and Multimodal Assessment:** This work contributes to the search for objective and digital markers in ADHD (Keshav et al., 2019; Teruel et al., 2024; Zhang et al., 2024). The RTS/SVM methodology employed here represents a sophisticated form of *digital phenotyping*. While methods such as AR/VR/games aim for ecological validity (Keshav et al., 2019; Teruel et al., 2024; Zheng et al., 2024; Kannen et al., 2024), the current paradigm offers experimental control to isolate temporal mechanisms. Convergence exists: performance in digital tasks correlates with clinical measures (Keshav et al., 2019; Teruel et al., 2024). The *fusion of multimodal data* improves classification (Zhang et al., 2024), suggesting that the visual findings presented here are part of a broader profile. The success of SVM in this study and of DNNs (Zhang et al., 2024) indicates the utility of *machine learning*. The sensitivity of RTS to SNR manipulation (p. 6) aligns with the importance of *cognitive load* in revealing deficits (Zhang et al., 2024; Teruel et al., 2024).

5. **Alpha Oscillations, Modulation Complexity, and Heterogeneity:** The relevance of the alpha band (p. 13) can be contrasted with the difficulty in *directly* modulating alpha power via tACS (Kannen et al., 2024), pointing to the complexity of oscillatory dynamics in ADHD. Processing *efficiency* linked to alpha may represent a more robust behavioral phenotype than neural power itself. Although the study finds high intra-group consistency (p. 18), the heterogeneity of ADHD (Faraone et al., 2015; Luo et al., 2019) and the variability in basic sensory processes (Jiang et al., 2024) indicate the need for future research on subtypes (Kannen et al., 2024). Conceptually, the difficulty in controlling attention, reflected in the oscillations, aligns with the idea that attentional bias influences perception (Rooney et al., 2024).

## Evaluation

**Technical Soundness and Data Support:** The manuscript (R2) demonstrates technical soundness within its scope. The methods are appropriate, and the analyses robustly support the conclusions regarding the

differential temporal signature in adult ADHD. The distinction between perceptual efficiency and neural mechanisms is made with scientific prudence (p. 16). Its value lies in providing *quantitative empirical parameters* for computational models of ADHD.

**Limitations and Theoretical Breadth:** The limitations (small non-medicated subgroup size, lack of medication details) are acknowledged and well-discussed (pp. 16-17, 20). They contextualize the inferences but do not diminish the importance of the central finding of the classifiable temporal signature. The discussion situates the findings within multiple relevant theoretical frameworks (oscillations, prediction, neuromodulation, digital assessment).

## Conclusion

This manuscript presents an original investigation, utilizing a pertinent methodology with theoretical relevance regarding the temporal bases of visual processing in adult ADHD. The authors have successfully demonstrated that the oscillatory dynamics of perceptual efficiency, revealed by random temporal sampling and quantified by machine learning, constitute a robust signature that differentiates individuals with ADHD from controls and reflects medication status. These findings provide empirical support for models that conceive of ADHD not merely as a static attention deficit, but as an alteration in the dynamic regulation of information processing, potentially linked to an atypical balance between prediction and sensory error. The work contributes to the understanding of ADHD as a condition affecting the *dynamic regulation* of the brain's interaction with a constantly changing world. The conclusions are well-supported by the data, the limitations are addressed with scientific integrity, and the potential for future research – including clinical applications as a biomarker and transdiagnostic investigations – is evident. This study demonstrates how rigorous psychophysics, combined with advanced computational analyses and a deep neuroscientific foundation, can advance our understanding of complex disorders such as ADHD. The manuscript, in its current form (R2), meets the publication criteria for PLOS ONE. I recommend its acceptance.

## References


1. Adamou, M., Fullen, T., & Jones, S. L. (2020). EEG for diagnosis of adult ADHD: A systematic review with narrative analysis. *Frontiers in Psychiatry*, *11*, 871. https://doi.org/10.3389/fpsyt.2020.00871
2. American Psychiatric Association. (2013). *Diagnostic and statistical manual of mental disorders* (5th ed.). American Psychiatric Publishing, Inc.
3. Arguin, M., Ferrandez, R., & Massé, J. (2021). Oscillatory visual mechanisms revealed by random temporal sampling. *Scientific Reports*, *11*(1), 21309. https://doi.org/10.1038/s41598-021-00642-y
4. Arguin, M., & Fortier-St-Pierre, S. (2023). *Spatiotemporal Letter Processing in Visual Word Recognition Uncovered by Perceptual Oscillations*. SSRN. https://papers.ssrn.com/abstract=4635714
5. Avisar, A., & Shalev, L. (2011). Sustained Attention and Behavioral Characteristics Associated with ADHD in Adults. *Applied Neuropsychology*, *18*(2), 107–116. https://doi.org/10.1080/09084282.2010.547706
6. Bates, M. E., & Lemay, E. P. (2004). The d2 Test of attention: construct validity and extensions in scoring techniques. *Journal of the International Neuropsychological Society*, *10*(3), 392–400.
7. Bellato, A., Arora, I., Kochhar, P., Hollis, C., & Groom, M. J. (2020). A systematic review of the effects of physical activity interventions on cognition in children and adolescents with ADHD. *Journal of Attention Disorders*, *24*(13), 1784–1806. https://doi.org/10.1177/1087054717707479
8. Bertrand Pilon, C., & Arguin, M. (2024). The processing of spatial frequencies through time in visual word recognition. *Scientific Reports*, *14*(1), 6628. https://doi.org/10.1038/s41598-024-57206-5
9. Bidwell, L. C., McClernon, F. J., & Kollins, S. H. (2011). Cognitive enhancers for the treatment of ADHD. *Pharmacology Biochemistry and Behavior*, *99*(2), 262–274. https://doi.org/10.1016/j.pbb.2011.05.002
10. Buzsáki, G. (2006). *Rhythms of the brain*. Oxford University Press.
11. Butzbach, M., Fuermaier, A. B. M., Aschenbrenner, S., Weisbrod, M., Tucha, L., & Tucha, O. (2019). Basic processes as foundations of cognitive impairment in adult ADHD. *Journal of Neural Transmission*, *126*(10), 1347–1362. https://doi.org/10.1007/s00702-019-02048-y
12. Chauvin, A., Worsley, K. J., Schyns, P. G., Arguin, M., & Gosselin, F. (2005). Accurate statistical tests for smooth classification images. *Journal of Vision*, *5*(9), 1. https://doi.org/10.1167/5.9.1
13. Clarke, A. R., Barry, R. J., & Johnstone, S. J. (2020). Resting state EEG power research in Attention-Deficit/Hyperactivity Disorder: A review update. *Clinical Neurophysiology*, *131*(7), 1463–1479. https://doi.org/10.1016/j.clinph.2020.02.002
14. Cohen, M. X. (2014). *Analyzing neural time series data: Theory and practice*. MIT Press.
15. Conners, C. K., Pitkanen, J., & Rzepa, S. R. (2011). Conners 3rd Edition (Conners 3; Conners 2008). In J. S. Kreutzer, J. DeLuca, & B. Caplan (Eds.), *Encyclopedia of Clinical Neuropsychology* (pp. 675–678). Springer. https://doi.org/10.1007/978-0-387-79948-3_1534

16. Content, A., Mousty, P., & Radeau, M. (1990). BRULEX. Une base de données lexicales informatisée pour le français écrit et parlé [BRULEX: A computerized lexical data base for the French language.]. *L'Année Psychologique*, *90*(4), 551–566.
17. Doyle, A. E., Faraone, S. V., Seidman, L. J., Willcutt, E. G., Nigg, J. T., Waldman, I. D., ... & Biederman, J. (2005). Are endophenotypes based on measures of executive functions useful for molecular genetic studies of ADHD? *Journal of Child Psychology and Psychiatry*, *46*(7), 774–803. https://doi.org/10.1111/j.1469-7610.2004.00396.x
18. Faraone, S. V., Asherson, P., Banaschewski, T., Biederman, J., Buitelaar, J. K., Ramos-Quiroga, J. A., ... & Franke, B. (2015). Attention-deficit/hyperactivity disorder. *Nature Reviews Disease Primers*, *1*, 15020. https://doi.org/10.1038/nrdp.2015.20
19. Friston, K. (2005). A theory of cortical responses. *Philosophical Transactions of the Royal Society B: Biological Sciences*, *360*(1456), 815–836. https://doi.org/10.1098/rstb.2005.1622
20. Gaillard, C., & Ben Hamed, S. (2022). The neural bases of spatial attention and perceptual rhythms. *European Journal of Neuroscience*, *55*(11–12), 3209–3223. https://doi.org/10.1111/ejn.15386
21. Gonzalez-Gadea, M. L., Señoran, L., Halliburton, A., Arnedo, M., García-Cordero, I., De Lillo, M., ... & Ibáñez, A. (2015). Predictive coding in autism spectrum disorder and attention deficit hyperactivity disorder. *Journal of Neurophysiology*, *114*(5), 2625–2636. https://doi.org/10.1152/jn.00206.2015
22. Gropper, R. J., Gotlieb, H., Kronitz, R., & Tannock, R. (2014). Working memory training in college students with ADHD or LD. *Journal of Attention Disorders*, *18*(4), 331–345. https://doi.org/10.1177/1087054712441772
23. Haigh, A., & Buckby, B. (2024). Rhythmic Attention and ADHD: A Narrative and Systematic Review. *Applied Psychophysiology and Biofeedback*, *49*(2), 185–204. https://doi.org/10.1007/s10484-023-09624-z
24. Hartanto, T. A., Krafft, C. E., Iosif, A. M., & Schweitzer, J. B. (2016). A trial-by-trial analysis reveals more intense physical activity is associated with better cognitive control performance in children with ADHD. *Child Neuropsychology*, *22*(5), 618–626. https://doi.org/10.1080/09297049.2015.1044511
25. Hearst, M. A., Dumais, S. T., Osuna, E., Platt, J., & Scholkopf, B. (1998). Support vector machines. *IEEE Intelligent Systems and their Applications*, *13*(4), 18–28. https://doi.org/10.1109/5254.708428
26. Hesson, J., & Fowler, K. (2018). Prevalence and Correlates of Self-Reported ADD/ADHD in a Large National Sample of Canadian Adults. *Journal of Attention Disorders*, *22*(2), 191–200. https://doi.org/10.1177/1087054715587837
27. Hohwy, J. (2013). *The predictive mind*. Oxford University Press.
28. Jiang, P., Kent, C., & Rossiter, J. (2024). Towards sensory substitution and augmentation: Mapping visual distance to audio and tactile frequency. *PLOS ONE*, *19*(3), e0299213. https://doi.org/10.1371/journal.pone.0299213
29. Kannen, K., Rasbach, J., Fantazi, A., Wiebe, A., Selaskowski, B., Asché, L., Aslan, B., Lux, S., Herrmann, C. S., Philipsen, A., & Braun, N. (2024). Alpha modulation via transcranial alternating current stimulation in adults

with attention-deficit hyperactivity disorder. *Frontiers in Psychology*, *14*, 1280397. https://doi.org/10.3389/fpsyg.2023.1280397
30. Keshav, N. U., Vogt-Lowell, K., Vahabzadeh, A., & Sahin, N. T. (2019). Digital Attention-Related Augmented-Reality Game: Significant Correlation between Student Game Performance and Validated Clinical Measures of Attention-Deficit/Hyperactivity Disorder (ADHD). *Children*, *6*(6), 72. https://doi.org/10.3390/children6060072
31. Khan, M. U., & Aslani, P. (2021). Exploring factors influencing initiation, implementation and discontinuation of medications in adults with ADHD. *Health Expectations*, *24*(S1), 82–94. https://doi.org/10.1111/hex.13209
32. Kim, S., Liu, Z., Glizer, D., Tannock, R., & Woltering, S. (2014). Adult ADHD and working memory: Neural evidence of impaired encoding. *Clinical Neurophysiology*, *125*(8), 1596–1603. https://doi.org/10.1016/j.clinph.2013.11.040
33. Keitel, C., Ruzzoli, M., Dugué, L., Busch, N. A., & Benwell, C. S. Y. (2022). Rhythms in cognition: The evidence revisited. *European Journal of Neuroscience*, *55*(11–12), 2991–3009. https://doi.org/10.1111/ejn.15212
34. Kleiner, M., Brainard, D., & Pelli, D. (2007). What's new in Psychtoolbox-3? *Perception*, *36*(ECVP Abstract Supplement), 14.
35. Klimesch, W. (2012). Alpha-band oscillations, attention, and controlled access to stored information. *Trends in Cognitive Sciences*, *16*(12), 606–617. https://doi.org/10.1016/j.tics.2012.10.007
36. Koehler, S., Lauer, P., Schreppel, T., Jacob, C., Heine, M., Boreatti-Hümmer, A., ... & Fallgatter, A. J. (2009). Increased EEG power density in alpha and theta bands in adult ADHD patients. *Journal of Neural Transmission*, *116*(1), 97–104. https://doi.org/10.1007/s00702-008-0130-8
37. Konrad, K., Neufang, S., Fink, G. R., & Herpertz-Dahlmann, B. (2007). Long-term effects of methylphenidate on neural networks associated with executive attention in children with ADHD: results from a longitudinal functional MRI study. *Journal of the American Academy of Child and Adolescent Psychiatry*, *46*(12), 1633–1641. https://doi.org/10.1097/chi.0b013e3181573177
38. Lévesque, M., & Arguin, M. (2024). The oscillatory features of visual processing are altered in healthy aging. *Frontiers in Psychology*, *15*, 1323493. https://doi.org/10.3389/fpsyg.2024.1323493
39. Luo, Y., Weibman, D., Halperin, J. M., & Li, X. (2019). A Review of Heterogeneity in Attention Deficit/Hyperactivity Disorder (ADHD). *Frontiers in Human Neuroscience*, *13*, 42. https://doi.org/10.3389/fnhum.2019.00042
40. Mamiya, P. C., Arnett, A. B., & Stein, M. A. (2021). Precision Medicine Care in ADHD: The Case for Neural Excitation and Inhibition. *Brain Sciences*, *11*(1), 91. https://doi.org/10.3390/brainsci11010091
41. Mazaheri, A., Fassbender, C., Coffey-Corina, S., Hartanto, T. A., Schweitzer, J. B., & Mangun, G. R. (2014). Differential oscillatory electroencephalogram between Attention-Deficit/Hyperactivity Disorder subtypes and typically developing adolescents. *Biological Psychiatry*, *76*(5), 422–429. https://doi.org/10.1016/j.biopsych.2013.10.019
42. McAvinue, L. P., Vangkilde, S., Johnson, K. A., Habekost, T., Kyllingsbæk, S., Bundesen, C., ... & Robertson, I. H. (2015). A componential analysis of

visual attention in children with ADHD. *Journal of Attention Disorders*, *19*(10), 882–894. https://doi.org/10.1177/1087054712465335

43. Micoulaud-Franchi, J. A., Lopez, R., Cermolacce, M., Vaillant, F., Péri, P., Boyer, L., ... & Vion-Dury, J. (2015). Sensory gating in adult ADHD: Event-related potential and perceptual experience reports comparisons with schizophrenia. *Biological Psychology*, *107*, 16–23. https://doi.org/10.1016/j.biopsycho.2015.02.009
44. Milanova, G., & Arguin, M. (2024, May 25). *The processing sequence in visual object recognition investigated by temporal sampling* [Poster presentation]. Annual conference of the Société Québécoise pour la Recherche en Psychologie, Drummondville, Québec, Canada.
45. Mostert, J. C., Onnink, A. M. H., Klein, M., Dammers, J., Harneit, A., Schulten, T., ... & Buitelaar, J. K. (2015). Cognitive heterogeneity in adult attention deficit/hyperactivity disorder: A systematic analysis of neuropsychological measurements. *European Neuropsychopharmacology*, *25*(11), 2062–2074. https://doi.org/10.1016/j.euroneuro.2015.08.014
46. Owens, A. P., Dargue, N., Howard, C., Mulvey, M. R., Campos, D., Ropar, D., ... & Cook, J. L. (2021). Interoceptive training to target anxiety in autistic adults (ADIE): A single-center, superiority randomized controlled trial. *eClinicalMedicine*, *39*, 101042. https://doi.org/10.1016/j.eclinm.2021.101042
47. Polanczyk, G., de Lima, M. S., Horta, B. L., Biederman, J., & Rohde, L. A. (2007). The worldwide prevalence of ADHD: a systematic review and metaregression analysis. *American Journal of Psychiatry*, *164*(6), 942–948. https://doi.org/10.1176/ajp.2007.164.6.942
48. Pretus, C., Ramos-Quiroga, J. A., Richarte, V., Corrales, M., Picado, M., Carmona, S., ... & Vilarroya, O. (2017). Time and psychostimulants: Opposing long-term structural effects in the adult ADHD brain. A longitudinal MR study. *European Neuropsychopharmacology*, *27*(12), 1238–1247. https://doi.org/10.1016/j.euroneuro.2017.10.040
49. Romei, V., Gross, J., & Thut, G. (2012). Sounds reset rhythms of visual cortex and corresponding human visual perception. *Current Biology*, *22*(9), 807–813. https://doi.org/10.1016/j.cub.2012.03.025
50. Rooney, T., Sharpe, L., Todd, J., Livesey, E., & Colagiuri, B. (2024). Understanding the Role of Expectancy, Anticipatory Anxiety, and Attention Bias in Nocebo Hyperalgesia: A Gaze-Contingent Attention Bias Modification Study. *The Journal of Pain*, *25*(4), 946–961. https://doi.org/10.1016/j.jpain.2023.10.016
51. Rubia, K. (2018). Cognitive neuroscience of Attention Deficit Hyperactivity Disorder (ADHD) and its clinical translation. *Frontiers in Human Neuroscience*, *12*, 100. https://doi.org/10.3389/fnhum.2018.00100
52. Samengo, I., Kay, J. W., & Hasenstaub, A. (2022). Bridging physiological and perceptual views of autism by means of sampling-based Bayesian inference. *PLOS Computational Biology*, *18*(1), e1009775. https://doi.org/10.1371/journal.pcbi.1009775
53. Sara, S. J., & Bouret, S. (2012). Orienting and reorienting: the locus coeruleus mediates cognition through arousal. *Neuron*, *76*(1), 130–141. https://doi.org/10.1016/j.neuron.2012.09.011

54. Shrout, P. E., & Fleiss, J. L. (1979). Intraclass correlations: Uses in assessing rater reliability. *Psychological Bulletin*, *86*(2), 420–428. https://doi.org/10.1037/0033-2909.86.2.420
55. Sikström, S., & Söderlund, G. (2007). Stimulus-dependent dopamine release in attention-deficit/hyperactivity disorder. *Psychological Review*, *114*(4), 1047–1075. https://doi.org/10.1037/0033-295X.114.4.1047
56. Sohn, H., Kim, I., Lee, W., Peterson, B. S., Hong, H., Chae, J. H., ... & Jeong, J. (2010). Linear and non-linear EEG analysis of adolescents with attention-deficit/hyperactivity disorder during a cognitive task. *Clinical Neurophysiology*, *121*(11), 1863–1870. https://doi.org/10.1016/j.clinph.2010.04.012
57. Song, P., Zha, M., Yang, Q., Zhang, Y., Li, X., & Rudan, I. (2021). The prevalence of adult attention-deficit hyperactivity disorder: A global systematic review and meta-analysis. *Journal of Global Health*, *11*, 04009. https://doi.org/10.7189/jogh.11.04009
58. Suthaharan, S. (2016). Support Vector Machine. In S. Suthaharan (Ed.), *Machine Learning Models and Algorithms for Big Data Classification: Thinking with Examples for Effective Learning* (pp. 207–235). Springer. https://doi.org/10.1007/978-1-4899-7641-3_9
59. Swanson, J., Baler, R. D., & Volkow, N. D. (2011). Understanding the effects of stimulant medications on cognition in individuals with attention-deficit hyperactivity disorder: a decade of progress. *Neuropsychopharmacology*, *36*(1), 207–226. https://doi.org/10.1038/npp.2010.160
60. ter Huurne, N., Onnink, M., Kan, C., Franke, B., Buitelaar, J., & Jensen, O. (2013). Behavioral consequences of aberrant alpha lateralization in attention-deficit/hyperactivity disorder. *Biological Psychiatry*, *74*(3), 227–233. https://doi.org/10.1016/j.biopsych.2012.11.009
61. Teruel, M. A., Sanchis, J., Ruiz-Robledillo, N., Albaladejo-Blázquez, N., Ferrer-Cascales, R., & Trujillo, J. (2024). Measuring attention of ADHD patients by means of a computer game featuring biometrical data gathering. *Heliyon*, *10*(4), e26555. https://doi.org/10.1016/j.heliyon.2024.e26555
62. Tucha, L., Fuermaier, A. B. M., Koerts, J., Buggenthin, R., Aschenbrenner, S., Weisbrod, M., ... & Tucha, O. (2017). Sustained attention in adult ADHD: Time-on-task effects of various measures of attention. *Journal of Neural Transmission*, *124*(S1), 39–53. https://doi.org/10.1007/s00702-015-1472-0
63. van Dongen-Boomsma, M., Lansbergen, M. M., Bekker, E. M., Kooij, J. J. S., van der Molen, M. W., Kenemans, J. L., & Buitelaar, J. K. (2010). Relation between resting EEG to cognitive performance and clinical symptoms in adults with attention-deficit/hyperactivity disorder. *Neuroscience Letters*, *469*(1), 102–106. https://doi.org/10.1016/j.neulet.2009.11.064
64. VanRullen, R. (2018). Attention Cycles. *Neuron*, *99*(4), 632–634. https://doi.org/10.1016/j.neuron.2018.08.009
65. Volkow, N. D., Wang, G. J., Kollins, S. H., Wigal, T. L., Newcorn, J. H., Telang, F., ... & Swanson, J. M. (2009). Evaluating dopamine reward pathway in ADHD: Clinical implications. *JAMA*, *302*(10), 1084–1091. https://doi.org/10.1001/jama.2009.1308

66. Volkow, N. D., Wang, G. J., Tomasi, D., & Baler, R. D. (2011). The Stimulant Medication Outcome Pathway: Dopamine Reward Pathway Hypofunction in ADHD. *Molecular Psychiatry*, *16*(11), 1147–1154. https://doi.org/10.1038/mp.2011.95
67. Wang, G. J., Volkow, N. D., Wigal, T., Kollins, S. H., Newcorn, J. H., Telang, F., ... & Swanson, J. M. (2013). Long-Term Stimulant Treatment Affects Brain Dopamine Transporter Level in Patients with Attention Deficit Hyperactive Disorder. *PLOS ONE*, *8*(5), e63023. https://doi.org/10.1371/journal.pone.0063023
68. Zhang, K. F., Yeh, S. C., Wu, E. H. K., Xu, X., Tsai, H. J., & Chen, C. C. (2024). Fusion of Multi-Task Neurophysiological Data to Enhance the Detection of Attention-Deficit/Hyperactivity Disorder. *IEEE Journal of Translational Engineering in Health and Medicine*, *12*, 668–674. https://doi.org/10.1109/JTEHM.2024.3435553
69. Zheng, Q., Kei, K. T., Chiu, K. Y., & Shum, K. K. M. (2024). Study protocol of a randomised controlled trial of the effects of near-infrared spectroscopy neurofeedback training coupled with virtual reality technology in children with ADHD. *BMJ Open*, *14*(1), e093183. https://doi.org/10.1136/bmjopen-2024-093183
