# Supplementary material for: Visual processing oscillates differently through time for adults with ADHD
Source: PLoS One. 2025 Sep 2;20(9):e0310605. doi: 10.1371/journal.pone.0310605 (PMC12404544; doi:10.1371/journal.pone.0310605)
Supplement: Attachment — Submitted filename: Response to reviewers.docx [file pone.0310605.s002.docx]

We have examined the comments, questions and suggestions which arose from the review of the manuscript. In what follows, we will address the issues which were raised. Each change made to the manuscript is noted below along with its page number.

We hope that you will find our responses and manuscript changes satisfactory.

Best regards,

The authors

Pénélope Pelland-Goulet, Martin Arguin, Hélène Brisebois & Nathalie Gosselin

# General editor suggestions

**Q1. Please ensure that your manuscript meets PLOS ONE’s style requirements, including those for file naming. The PLOS ONE style templates can be found at**

[**https://journals.plos.org/plosone/s/file?id=wjVg/PLOSOne_formatting_sample_main_body.pdf**](https://journals.plos.org/plosone/s/file?id=wjVg/PLOSOne_formatting_sample_main_body.pdf)

[**https://journals.plos.org/plosone/s/file?id=ba62/PLOSOne_formatting_sample_title_authors_affiliations.pdf**](https://journals.plos.org/plosone/s/file?id=ba62/PLOSOne_formatting_sample_title_authors_affiliations.pdf)

A1. We have thoroughly checked the style/formatting requirements and that the present resubmission is in accordance. Slight formatting errors in affiliations were corrected (p. 1; lines 7-15).

We have also made minor corrections to figure 1 (enhanced visibility corrected the lowercase first word letter (“Litre” became “litre”) and to figures 3, 4, and 6’s titles. To favour format uniformity across all figures, we reupdated them all.

**Q2. Please include your full ethics statement in the ‘Methods’ section of your manuscript file. In your statement, please include the full name of the IRB or ethics committee who approved or waived your study, as well as whether or not you obtained informed written or verbal. If consent was waived for your study, please include this information in your statement as well.**

A2. The full ethics statement may be found on p. 5 (lines 99-103).

“Consent was also needed from a parent or guardian for participants under 18 years of age to enroll in the study. Data collection took place between January 17, 2021 and October 19, 2021. The study was approved by the Comité d’Éthique de la Recherche en Éducation et Psychologie (Education and Psychology Research Ethics Committee) of Université de Montréal and the Comité d'Éthique de la Recherche (Research Ethics Committee) of Montmorency College prior to any participant recruitment.”

**Q3. Please review your reference list to ensure that it is complete and correct. If you have cited papers that have been retracted, please include the rationale for doing so in the manuscript** **text, or remove these references and replace them with relevant current references. Any changes to the reference list should be mentioned in the rebuttal letter that accompanies your revised manuscript. If you need to cite a retracted article, indicate the article’s retracted status in the Reference list and also include a citation and full reference for the retraction notice.**

A3. We have done so. We also searched each article cited in our manuscript on the Retraction Watch Database (https://retractiondatabase.org/RetractionSearch.aspx) and found no match. for any of the references. Finally, we checked whether errata/corrections have been published for each cited article and found none.

Finally, please note that we have also added the following citations in the revised manuscript (all checked as above).

Methods:

p. 7; line 144 : Romei V, Gross J, Thut G. Sounds reset rhythms of visual cortex and corresponding human visual perception. Curr Biol. 2012 May 8;22(9):807-13. doi: 10.1016/j.cub.2012.03.025. Epub 2012 Apr 12. PMID: 22503499; PMCID: PMC3368263.

Discussion:

p. 16 & 17; lines 381 & 385 : Swanson J, Baler RD, Volkow ND. Understanding the effects of stimulant medications on cognition in individuals with attention-deficit hyperactivity disorder: a decade of progress. Neuropsychopharmacology. 2011 Jan;36(1):207-26. doi: 10.1038/npp.2010.160. Epub 2010 Sep 29. PMID: 20881946; PMCID: PMC3055506.

p. 17; line 385 : Bidwell LC, McClernon FJ, Kollins SH. Cognitive enhancers for the treatment of ADHD. Pharmacol Biochem Behav. 2011 Aug;99(2):262-74. doi: 10.1016/j.pbb.2011.05.002. Epub 2011 May 10. PMID: 21596055; PMCID: PMC3353150.

In p. 17; line 405, have also removed one reference (Milanova, Singh & Arguin, in preparation, Université de Montréal (previously #46)) and replaced it with the following references:

Milanova G & Arguin M. The processing sequence in visual object recognition investigated by temporal sampling. Communication. Annual conference of the Société Québécoise pour la Recherche en Psychologie (SQRP). 25 mai 2024, Drummondville, Québec, Canada.

Bertrand Pilon C & Arguin M. Le traitement des fréquences spatiales à travers le temps dans la reconnaissance des mots écrits. Communication. Annual conference of the Société Québécoise pour la Recherche en Psychologie (SQRP). 25 mai 2024, Drummondville, Québec, Canada.

**Q4. Additional editor comments: The authors need some detailed explanation of the medical assessments section and add more discussion sections as the reviewers suggested.**

A4. More information regarding medical assessment and discussion sections have been added. These are described in detail in our replies to reviewers.

# Reviewer #1

**Technical Soundness and Rigor:**

**Q1. The manuscript demonstrates high technical rigor, with each step of the methodology carried out exactly as outlined. The authors have implemented each procedure consistently and with clear adherence to established standards in the field.**

**Q2. The analytical methods are appropriately selected and well-executed, ensuring accurate interpretation of the findings.**

**Data Alignment with Conclusions:**

**Q3. The conclusions are directly supported by the data, with clear evidence presented for each claim. Each result is systematically connected to the corresponding data, making the conclusions reliable and well-founded.**

**Q4. The sample size and data quality lend strong credibility to the study, and the statistical analyses applied are appropriate, further substantiating the authors’ conclusions.**

A1-4. We appreciate the favorable evaluation of our methods.

**Additional Minor Comments (if any):**

**Q5. Although the manuscript meets high standards of technical precision, minor clarifications, such as an expanded explanation of [specific section], may further enhance clarity for readers.**

A5. Any methodological detail that we found was missing from the initial manuscript has been added in the present revision (see p. 5; line 116, p. 7; lines 144-146). Slight reformulations were also made to improve clarity in this section (see p. 6; lines 136, 138, p. 7; lines 147, 152 & 156, p. 9; lines 192, 193 & 199, p. 9; line 200, p. 11; line 245).

# Reviewer #2

**Strengths:**

**Q1. This study creatively combines random temporal sampling techniques and machine learning algorithms to explore differences in visual processing efficiency between individuals with ADHD and neurotypical controls. This approach not only reveals potential neural oscillatory features but also demonstrates its clinical application potential.**

**Q2. The high classification accuracy of machine learning (91.8%) highlights the effectiveness of the research methodology and provides strong evidence supporting the feasibility of oscillatory features as biomarkers for ADHD.**

**Q3. The study conducts an in-depth comparison of ADHD individuals and further analyzes differences between medicated and non-medicated participants, providing a solid foundation for future research on medication effects.**

**Q4. By integrating techniques and knowledge from neuroscience, psychology, and computer science, this research holds significant interdisciplinary value and is likely to attract attention from researchers across multiple fields.**

A1-4. Thank you for these positive comments on the manuscript and methods.

**Limitations and Suggestions for Improvement:**

**Q5. The sample size is relatively small (49 participants), particularly the group of non-medicated ADHD participants, which only includes 6 individuals. This limitation may affect the generalizability of the results. It is recommended to expand the sample size in future studies to enhance the external validity of the findings.**

A5. It is true that the sample size may seem limited. However, this sample size is relatively large for studies of this kind (e.g., 10 participants in Wang et al., 2011; 41 and 59 participants for two experiments in Blais et al., 2012; 19 participants in Wutz, Melcher & Samaha, 2018).

Moreover, as noted in the manuscript, the classification of data patterns according to the presence/absence of ADHD or the use of medication or not in ADHD participants along with a leave-one-out cross validation procedure constitutes a fairly robust support for the generalizability of our findings. Specifically, on every iteration, the classifier had to learn a correspondence pattern between the data patterns and the classification criteria for all participants but one, to then be tested on the data pattern that had been left out from the learning phase. Thus, to be successful, the mapping learned by the classifier had to remain valid for the test pattern for it to be successful For this reason, we maintain that the high classification rates obtained (up to 100%) offer in this study suggest that the discriminant information identified by the classifier for the “ADHD vs control” and the “medication vs not” problems is quite generalizable.

Wang HF, Friel N, Gosselin F, Schyns PG. Efficient bubbles for visual categorization tasks. Vision Res. 2011 Jun 21;51(12):1318-23. doi: 10.1016/j.visres.2011.04.007. Epub 2011 Apr 16. PMID: 21524660.

Blais C, Roy C, Fiset D, Arguin M, Gosselin F. The eyes are not the window to basic emotions. Neuropsychologia. 2012 Oct;50(12):2830-2838. DOI: 10.1016/j.neuropsychologia.2012.08.010. PMID: 22974675.

Wutz A, Melcher D, Samaha J. Frequency modulation of neural oscillations according to visual task demands. Proc Natl Acad Sci U S A. 2018 Feb 6;115(6):1346-1351. doi: 10.1073/pnas.1713318115. Epub 2018 Jan 22. PMID: 29358390; PMCID: PMC5819398.

**Q6. The study does not specify the types and dosages of stimulant medications used by participants, which could significantly influence the results. Future research should collect and analyze these details to assess the differential impacts of various medications on neural oscillations.**

A6. It is true that the exact type of medication and dosage used would be relevant information. Unfortunately, we did not ask the questions relevant to this issue in our General information questionnaire. We will make sure we do so in future studies. We should nevertheless emphasize that even without medication type/dosage information, it was possible to classify ADHD participants according to their use of medication or not with high accuracy. We take this to suggest that we were able to capture a common alteration of brain function that is exerted by the medications used in our ADHD sample, regardless of the exact medication type/dosage. This issue is further addressed on p. 16-17 (lines 379-386).

Relevant to the topic of medication are also lines 439-446 (p. 19-20) which were added in response to Question #9.

**Q7. Were ADHD diagnoses based on medical assessments? Were participants diagnosed in early childhood? Clarifying these aspects is crucial to understanding the heterogeneity within the study population.**

A7. We agree that these informations are relevant.

We do have information as to the profession of the persons who emitted the ADHD diagnosis and made sure that they are from professions abilitated to do so. We added those professions to the sample description. We address the issue in the revised manuscript on p. 4 (lines 88-90).

Unfortunately, however, we have no information as to the age of diagnosis.

**Q8. While the diagnostic value of oscillatory features is demonstrated, the study lacks a thorough exploration of their association with specific neural mechanisms. It is suggested that future research further investigate the relationship between these features and the functions of specific brain regions.**

A8. We absolutely agree with this view and work is underway in our lab to study the way the perceptual oscillations demonstrated by the technique of random temporal sampling relate to brain activity.

We have made more explicit the need for explicit demonstrations of the relationship between perceptual oscillations and brain activity (see p. 16; lines 368-369).

**Q9. The article provides limited discussion on how oscillatory features could be translated into concrete clinical tools. Adding practical examples or potential application scenarios in clinical diagnostics and treatment monitoring would enhance the article's applicability.**

A9. We have added discussion of this topic on p. 19-20 (lines 434-446).

# Conclusion and Overall Evaluation

**This study demonstrates the potential of random temporal sampling techniques and machine learning in identifying neural oscillatory features associated with ADHD. It holds significant scientific value and clinical applicability. However, limitations such as the small sample size and insufficient details on stimulant usage restrict the generalizability of the conclusions.**

**The innovative study design and comprehensive data analysis lay a solid foundation for future research. I recommend the study for publication following appropriate revisions to the discussion section, and I look forward to seeing further studies that validate and expand upon these findings.**
