# Supplementary material for: Visual processing oscillates differently through time for adults with ADHD
Source: PLoS One. 2025 Sep 2;20(9):e0310605. doi: 10.1371/journal.pone.0310605 (PMC12404544; doi:10.1371/journal.pone.0310605)
Supplement: Attachment — Submitted filename: Response_to_Reviewers_Feb_2025.docx [file pone.0310605.s004.docx]

February 6, 2025

Editorial office

PLoS One

Object: Manuscript PONE-D-24-35136R1, by Pelland-Goulet et al.

We have examined the comments, questions and suggestions which arose from the latest review of the manuscript. In what follows, we will address the issues which were raised. Each change made to the manuscript is noted below along with its page number.

We hope that you will find our responses and manuscript changes satisfactory.

Best regards,

The authors

Pénélope Pelland-Goulet, Martin Arguin, Hélène Brisebois & Nathalie Gosselin

# Reviewer #2

**Suggestions for improvements**

**Q1. Expand Sample Size: Future studies should include larger and more diverse populations to enhance external validity. Collect Detailed Medication Data: Include information on medication types, dosages, and duration to better assess their effects on neural oscillations. Explore Neural Mechanisms: Conduct complementary neuroimaging studies to link the observed oscillatory patterns to specific brain regions and networks.**

Thank you for these propositions. We have added the elements listed in your comment in the discussion (p. 20, lines 467-471).

**Additional Comments**

**Q2. The manuscript is well-structured, with clear figures and thorough methodological details. However, a more extensive discussion of the practical challenges in translating oscillatory features into clinical tools would enhance its applicability.**

Indeed, there are still challenges to overcome before the technique can be used in clinical settings. We have added discussion of this topic (p. 20, lines 464-466).

**Q3. Consider discussing the potential impact of task demands and cognitive load on the observed oscillatory patterns. The manuscript provides a strong foundation for understanding visual processing differences in ADHD and demonstrates the potential of random temporal sampling for diagnostic and monitoring purposes. Addressing the noted limitations would further strengthen its contribution to the field.**

We have added a more in-depth discussion of these topics (p. 20, lines 451-464).

# Reviewer #3

**Comments**

**Q1. “line 30 temporal sampling: Could you describe it more definitely”**

We have added more details regarding the temporal sampling method (p. 2, lines 24-26).

**Q2. “line 42/44 TBR ?? dose it mean theta/beta ratio??,please remark it clearly”**

Thank you for bringing this to our attention. We have clarified the exact wording of the TBR abbreviation (p. 2, lines 43-44).

**Q3. “in the section of material and method can you mention the classification of neurotypical control , or it just the same type group of diagnosis? if possible , what is the presentation of normal people group on temporal sampling and could you mention it in your article?”**

We have clarified the way in which participants were assigned to either the neurotypical or ADHD group (p. 4, lines 89-90).

As for the description of neurotypical participants group, we already describe in detail their results in the manuscript and there is no important information that was left out. It is not clear from R3’s comment what should be added in this regard.

**Q4. “line 402 for the medication vs none problem?? does it mean none stimulants medication? if it is, please modify it more clearly”**

We have revised the paragraph to clarify what data is discussed in this section (p. 17, line 407; p. 18; lines 413-417).
