# Supplementary material for: Visual processing oscillates differently through time for adults with ADHD
Source: PLoS One. 2025 Sep 2;20(9):e0310605. doi: 10.1371/journal.pone.0310605 (PMC12404544; doi:10.1371/journal.pone.0310605)
Supplement: Attachment — Submitted filename: Response_to_Reviewers_May_2025.docx [file pone.0310605.s005.docx]

May 9, 2025

Editorial office

PLoS One

Object: Manuscript PONE-D-24-35136R1, by Pelland-Goulet et al.

We have examined the comments, questions and suggestions which arose from the latest review of the manuscript. In what follows, we will address the issues which were raised. Each change made to the manuscript is noted below along with its page number.

We hope that you will find our responses and manuscript changes satisfactory.

Best regards,

The authors

Pénélope Pelland-Goulet, Martin Arguin, Hélène Brisebois & Nathalie Gosselin

# Reviewer #4

**Review Comments to the Author**

**Q1. The final sample included only 49 participants (26 neurotypical, 23 ADHD), which is very small, especially given the goal of distinguishing individuals based on subtle neurocognitive markers. This small size can lead to statistical noise being mistaken for true signal.**

A1. It is true that the sample size may seem limited. We also believe that for these markers to be used in clinical settings, the results would need to be replicated with different, larger samples, to ensure that the observations are truly generalizable across ADHD adults. However, this sample size is relatively large for studies investigating visual mechanisms using techniques similar to ours (e.g., 10 participants in Wang et al., 2011; 41 and 59 participants for two experiments in Blais et al., 2012; 19 participants in Wutz, Melcher & Samaha, 2018).

Moreover, as noted in the manuscript, the classification of data patterns according to the presence/absence of ADHD or the use of medication or not in ADHD participants along with a Leave-one-out cross validation procedure constitutes a fairly robust support for the generalizability of our findings. Specifically, on every iteration, the classifier had to learn the correspondence between the data patterns and the categories for classification for all participants but one, to then be tested on the data pattern that had been left out from the learning phase. Thus, to be successful, the mapping learned by the classifier had to remain valid for the test pattern for it to be successful. For this reason, we maintain that the high classification rates obtained (up to 100%) in this study suggest that the discriminant information identified by the classifier for the “ADHD vs control” and the “medication vs not” problems is rather generalizable.

Blais, C., Roy, C., Fiset, D., Arguin, M. & Gosselin, F. (2012). The eyes are not the window to basic emotions. Neuropsychologia, 50(12):2830-2838. DOI: 10.1016/j.neuropsychologia.2012.08.010.

Wang, H. F., Friel, N., Gosselin, F., Schyns, P. G. (2011). Efficient bubbles for visual categorization tasks. Vision Res, 51(12):1318-23. doi: 10.1016/j.visres.2011.04.007.

Wutz, A., Melcher, D. & Samaha, J. (2018) Frequency modulation of neural oscillations according to visual task demands. Proc Natl Acad Sci U S A, 115(6):1346-1351. doi: 10.1073/pnas.1713318115.

**Q2. The machine learning classifier achieved 91.8% accuracy using a subset of features, which is impressive. However, there is no indication of external validation, such as applying the model to a new dataset or performing a stratified k-fold cross-validation.**

A2. Regarding the method of cross-validation, it is true that we did not test the model on a brand-new dataset. However, we used a Leave-one-out (LOO) cross-validation method, which is capable of addressing much of the issues that can be addressed with stratified k-fold cross-validation (Yadav & Shukla, 2016; Sreedharan et al., 2023).

While the first classification problem compared groups of similar sample sizes (23 ADHD participants vs 26 neurotypical participants), it is true that the second classification problem compared unequal groups (6 non-medicated vs 17 medicated ADHD participants), which created an imbalance in the quantity of data representing each label in this classification task.

In the interest of ensuring maximal validity of our results, as per the suggestion of Reviewer 4, we tested the stratified k-fold cross-validation method (Prusty, Patnaik & Dash, 2022). However, the results were not satisfying: the classifier only reached 69.2% accuracy using all available data when predicting group (ADHD vs neurotypical) and 75.5% accuracy using all data when predicting medication usage among ADHD participants.

Our choice of the LOO method was made based on the observation that for small samples LOO is more appropriate, while k-fold is more appropriate for larger samples (Yadav & Shukla, 2016). Indeed, the LOO method ensures that a maximum of data is used during the training phase while testing the model on each individual participant when it was excluded from the training set (Sreedharan et al., 2023). In our case, even using a very small k (two folds) reduced the training set to rather small numbers, especially for the non-medicated group. These small samples seem to have been insufficient for the SVM to properly capture the crucial features by which groups differ. Indeed, according to Akbani, Kwek & Japkowicz (2004), undersampling the largest of the imbalanced samples (i.e. reducing its size to be more similar to that of the smallest sample) reduces the available information available for the model, limiting its accuracy.

Overall then, we maintain our use of the LOO cross-validation method, which appears as the most appropriate given the present sample. Data using the k-fold method is not reported given the sample size problem it runs into, as noted above.

In order to describe the classification results in greater detail, we have computed the sensitivity and specificity for both classifications problems at the stopping point of 90% or above classification performance. When participants were classified according to whether they have ADHD or not, we obtained a sensitivity of 96.2% and a specificity of 87%. For the medication classification, sensitivity was of 100%, with a specificity of 66.7%. These numbers are excellent, except for the specificity of the medication classification problem. The lower specificity of 66.7% for this problem may be attributed to the small number of non-medicated participants, which seems to have prevented the classifier to learn data patterns specifically associated with not using medication.

We have added the sensitivity and specificity indexes in the manuscript at p. 12, lines 282-283 (group classification), p. 14, lines 331-332 (medication classification) and p. 15, line 360; p. 16, lines 381-386 (general discussion).

Akbani, R., Kwek, S., Japkowicz, N. (2004). Applying Support Vector Machines to Imbalanced Datasets. In: Boulicaut, JF., Esposito, F., Giannotti, F., Pedreschi, D. (eds) Machine Learning: ECML 2004. ECML 2004. Lecture Notes in Computer Science, vol 3201. Springer, Berlin, Heidelberg. <https://doi.org/10.1007/978-3-540-30115-8_7>

Prusty, Sashikanta, Patnaik, Srikanta & Dash, Sujit Kumar. (2022). SKCV: Stratified K-fold cross-validation on ML classifiers for predicting cervical cancer. *Frontiers in Nanotechnology*, 4. <https://doi.org/10.3389/fnano.2022.972421>

Sreedharan, R., Prajapati, J., Engineer, P. & Prajapati, D. (2023). Leave-One-Out Cross-Validation in Machine Learning. Ethical Issues in AI for Bioinformatics and Chemoinformatics, 1^st^ edition. pp. 56-71. <http://dx.doi.org/10.1201/9781003353751-5>

Yadav, S. & Shukla, S. (2016). Analysis of k-Fold Cross-Validation over Hold-Out Validation on Colossal Datasets for Quality Classification. *2016 IEEE 6th International Conference on Advanced Computing (IACC)*, Bhimavaram, India, pp. 78-83, doi: 10.1109/IACC.2016.25.

**Q3. P-values are used throughout for statistical comparisons (e.g., for CAARS scores, gender balance), but no effect sizes are provided.**

A3. Thank you for this observation. We have added the effect sizes for statistical comparisons (ADHD vs neurotypical comparisons (p. 11, lines 252 to 260) and ADHD medicated vs ADHD non-medicated (p. 13-14, lines 311-318)).

# Reviewer #5

**Review comments to the Author**

**The manuscript, in its current form (R2), meets the publication criteria for PLOS ONE. I recommend its acceptance.**

We want to thank Reviewer 5 for taking the time to make such a thorough examination of our manuscript and providing in depth analysis of our results.

R5 asked for no additional modification to the manuscript.
